# Veterinary World reviewer acknowledgment 2022

**DOI:** 10.14202/vetworld.2023.246-249

**Published:** 2023-02-08

**Authors:** A. V. Sherasiya, Nazir

**Affiliations:** Veterinary World, Star, Gulshan Park, NH-8A, Chandrapur Road, Wankaner - 363621, Dist. Morbi, Gujarat, India

**Contributing reviewers**

Veterinary World editorial team would sincerely like to thank all of our reviewers who contributed to peer review for the journal in 2022.

**Table:** 

No.	Reviewer	Country	Reviewed Articles
1	A Aygun Aygun	Turkey	2
2	Á. Bodnár	Hungary	1
3	A. I. Patiu	Romania	2
4	Aamir Ali	Pakistan	1
5	Abdallah M.A. Hassane	Egypt	1
6	Abdelazeem M Algammal	Egypt	1
7	Abel Villa-Mancera	Mexico	4
8	Abid Aslam Maan	Pakistan	1
9	Abolfazl Hajibemani	Iran	1
10	Ahmad S. Kodous	India	1
11	Ahmed Ali Ali	Egypt	1
12	Ahmed S Abdoon	Egypt	2
13	Alberto Elmi	Italy	2
14	Alejandro Hidalgo	Chile	4
15	Alexandre Lamas	Spain	1
16	Alexandria P. Snider	USA	1
17	Alfredo Medrano	Mexico	2
18	Alireza Seidavi	Iran	1
19	Alok Kumar Dixit	India	1
20	Amitava Dey	India	1
21	Ana Cristina Afonso	Portugal	1
22	Ananda Raja Ramalingam	India	1
23	Andreia Garcês	Portugal	1
24	Andres Carvajal	Chile	1
25	Anna Didkowska	Poland	1
26	Anuj Ahuja	USA	1
27	Anut Chantiratikul	Thailand	4
28	Ariadna Flores Ortega	Mexico	1
29	Ashwani Kumar	India	1
30	Awatef Bejaoui	Tunisia	2
31	Balamurugan Vinayagamurthy	India	1
32	Baratang Alison Lubisi	South Africa	3
33	Bartosz Kieronczyk	Poland	1
34	Batoul Mohamed Izzularab	Egypt	1
35	Belgin Siriken	Turkey	3
36	Bemela Mawulom Tokofai	Togo	1
37	Breda Jakovac-Strajn	Slovenia	1
38	Buket Er Demirhan	Turkey	1
39	Chahrazed Belhout	Algeria	1
40	Chao-Nan Lin	China	1
41	Christian Bux	Italy	1
42	Chyer Kim	USA	1
43	Cord M. Brundage	USA	1
44	Dakshina Bisht	India	1
45	Daniel Diaz	Mexico	1
46	Deborah Nadal	Italy	1
47	Deepan Kishore	USA	1
48	Dehui Yin	China	1
49	Dhary Alewy Almashhadany	Iraq	1
50	Dhruv Nitinkumar Desai	USA	3
51	Dinah Seligsohn	Sweden	1
52	Diya Abdel Moemen El-Badry	Egypt	1
53	Doha Houssien Abou Baker	Egypt	1
54	Dominik Lagowski	Poland	3
55	Dr. Archana Jain	India	1
56	Eduardo Jorge Boeri	Argentina	4
57	Eimad dine Tariq Bouhlali	Morocco	1
58	Elisa M. Mazzaferro	USA	2
59	Eman Dhahir Arif	Iraq	1
60	Emiliano Lasagna	Italy	1
61	Emily PattersonKane	USA	1
62	Emma L. Fairbanks	Switzerland	1
63	Enrica Sozzi	Italy	1
64	Erma Safitri	Indonesia	2
65	Eva Furrow	USA	1
66	F. Birettoni	Italy	1
67	Fabrizio Bertelloni	Italy	2
68	Fakhr Mostafa Lashein	Egypt	1
69	Farida Khammar	Algeria	1
70	Fathiah Zakham	Finland	1
71	Federica Di Cesare	Italy	1
72	Fouad Kasim Mohammad	Iraq	6
73	Gabriele Maier	USA	1
74	Girija Regmi	USA	4
75	Gnanavel Venkatesan	India	1
76	Guang-You Yang	China	1
77	Gyanendra Gongal	India	1
78	Hans Van Van Loo	Belgium	1
79	Harvie P. Portugaliza	Philippines	2
80	Hasria Alang	Indonesia	3
81	Hassan A Hussein Hussein	Egypt	1
82	Hazem Shaheen	Egypt	1
83	Heba M. Salem	Egypt	1
84	HuaJi Qiu	China	4
85	Hua-Liang Li	China	1
86	Hudson A. Pinto	Brazil	1
87	Huseyin Yilmaz	Turkey	1
88	I. Loncaric	Austria	1
89	Iang Schroniltgen Rondon Barragan	Colombia	2
90	İlknur Dağ	Turkey	1
91	Indranil Samanta	India	1
92	Indrasen Chauhan	India	1
93	Inga Stadaliene	Lithuania	1
94	Ioana Matei	Romania	1
95	Ionel D. BONDOC	Romania	3
96	Ionela Hotea	Romania	1
97	Ipsita Mohanty	USA	3
98	Ismael Hernandez Avalos	Mexico	2
99	J. Werszko	Poland	1
100	Jacek Osek	Poland	1
101	Jamal Gharekhani	Iran	2
102	Jareerat Aiemsaard	Thailand	2
103	João Simões	Portugal	2
104	Joaquin Gadea	Spain	1
105	John Kagira	Kenya	1
106	Juan Busso	Argentina	1
107	K. S. Rashm	India	1
108	Kalman Imre	Romania	1
109	Kannan Thandavan Arthanari	India	2
110	Karim ElSabrout	Egypt	3
111	Kashif Prince	Pakistan	1
112	Katia da Silva Calabrese	Brazil	1
113	Keisuke Suganuma	Japan	1
114	kekungu puro	India	1
115	Ki Hyun Kim	Kosovo	1
116	Koushlesh Ranjan	India	1
117	Krpasha Govindasamy	India	1
118	Laxmi Narayan Sarangi	India	1
119	Lenin Lenin Aguirre Riofrio	Ecuador	2
120	Li Peng Tan	Malaysia	1
121	Liben Chen	USA	1
122	Liga Kovalcuka	Latvia	1
123	Liliana Aguilar-Marcelino	Mexico	3
124	Luit Barkalita	Belgium	2
125	Lukasz Migdal	Poland	1
126	Lus Cardoso	Portugal	1
127	M Zamri Saad	Malaysia	2
128	Mabel Kamweli Aworh	Nigeria	2
129	Mahamudul Hasan	Bangladesh	1
130	Mahmoud Mohey Elhaig	Egypt	2
131	Mai Mohammed Abuowarda	Egypt	1
132	Majid Mottaghitalab	Iran	2
133	Maria de los Angeles GomezMorales	Italy	1
134	María Denisse Montoya-Flores	Mexico	1
135	María Teresa Cubo	Spain	1
136	Mário Manuel Dinis Ginja	Portugal	1
137	Marizvikuru Mwale	South Africa	1
138	Marwa Mohamed Attia	Egypt	1
139	Maryam Dadar	Iran	2
140	Masato Nitta	Japan	1
141	Matheus S. Monteiro	Brazil	1
142	Md. Ahaduzzaman	Bangladesh	2
143	Md. Tanvir Rahman	Bangladesh	1
144	Mehdi Shamsaie Mehrgan	Iran	1
145	Michelle Natividad	Philippines	1
146	Mirosław Mariusz Michalski	Poland	3
147	Mohamed Abdo Rizk	Japan	1
148	Mohammed A. E.	Egypt	1
149	Mohammed Fouad El Basuini	Egypt	1
150	Mohammed Hocine benaissa	Algeria	1
151	Mohanned Alhussien	Germany	1
152	Monika Olech	Poland	1
153	Mostafa F.N. Abushahba	USA	4
154	mosua tavassoli	Iran	1
155	Muawuz Ijaz	Pakistan	1
156	Muhammad Abdul Basit	Pakistan	1
157	Muhammad Arfan Zaman	Pakistan	1
158	Muhammad Asif Arain	Pakistan	1
159	Muhammad Usman	Pakistan	1
160	Murat Sevik	Turkey	3
161	N.K. Abd El-Aziz	Egypt	1
162	Nagah Arafat	Egypt	1
163	Nazarudin M.F.	Malaysia	1
164	Neil A. Mabbott	UK	1
165	Nicholas Johnson	United Kingdom	1
166	Nor Yasmin Abd Rahaman	Malaysia	1
167	Nuh Kilic	Turkey	4
168	Nur Azira Tukiran	Malaysia	1
169	Nurul Huda	Malaysia	1
170	Ola Hassanin	Egypt	1
171	OlaFadunsin Shola David	Nigeria	2
172	Omar Maamouri	Tunisia	1
173	Panagiotis E Simitzis	Greece	6
174	Pär Ewing	Sweden	1
175	Parag Nigam	India	1
176	Pavan Kumar Cherukuri	USA	1
177	Pie Ntampaka	Rwanda	1
178	Pierre Becker	Belgium	1
179	Pınar Şanlıbaba;	Turkey	3
180	Piotr Szweda	Poland	1
181	Prapansak Srisapoome	Thailand	2
182	Priyanka Uppe	India	1
183	Prof ElSayed Eldeeb	Egypt	3
184	Prof. Khaled Hafez, attia	Egypt	1
185	R.Umaya Suganthi	India	4
186	Raquel Salazar Lugo	Venezuela	2
187	Ratchaneegorn Mapanao	Thailand	1
188	Remil L. Galay	Philippines	1
189	René Renzhammer	Austria	1
190	Roberto D. Moyano	Argentina	1
191	Rodrigo Alberto Jerez Ebensperger	Spain	2
192	Rouaa Daou	Lebanon	1
193	Ruben Pérez	Uruguay	1
194	Rute Canejo-Teixeira	Portugal	1
195	Rute M Noiva	Portugal	1
196	Ruttayaporn Ngasaman	Thailand	1
197	S Mathan Kumar	Oman	1
198	Sabeena Sasidharan Pillai	Nepal	1
199	Sabrina Sheila Greening	New Zealand	1
200	Salma Bessalah	Tunisia	1
201	Sandrine Musa	Germany	1
202	Saw Bawm	Morocco	2
203	Shaimaa Ali Selim	Egypt	1
204	Shekhar Ramchandra Bhagwat	India	2
205	Sherin R. Rouby	Egypt	1
206	Socorro Retana-Marquez	Mexico	1
207	Sofía Grecco	Uruguay	1
208	Sorin Daniel Dan	Romania	1
209	süleyman çilek	Turkey	4
210	Sumera Sajjad	Pakistan	1
211	Suresh H Basagoudanavar	India	3
212	Susan A. McCoard	New Zealand	1
213	Takalani Judas Mpofu	South Africa	1
214	Takeshi Shimogiri	Japan	1
215	Tanko Polycarp Nwunuji	Nigeria	3
216	Tapan Bhattacharyya	United Kingdom	1
217	Tariq Jamil	Germany	2
218	ThankGod E. Onyiche	Nigeria	1
219	Vanesa Ruiz	Argentina	1
220	Vanessa S. Cruz	Brazil	2
221	Varij Nayan	India	2
222	Ved Prakash	India	1
223	Veronica Risco-Castillo	France	1
224	Vincentius Arca Testamenti	Indonesia	1
225	Vukka Chengalva Rayulu	India	1
226	Widya Paramita Lokapirnasari	Indonesia	4
227	Win Surachetpong	Thailand	1
228	Xueyong Zhang	China	1
229	Y. Liu	China	1
230	Ya-Ching Lin	Taiwan	1
231	Yaser Hamadeh Tarazi	Jordan	1
232	Yasmine Hasanine Tartor	Egypt	2
233	Yosra AMdouni	Tunisia	1
234	Yuqing Liu	China	1
235	Zahid Naseer	Pakistan	1
236	Zhenlei Zhou	China	2
237	Zhi Cao	China	1
238	Zhong Peng	China	1
239	Zuhair Ismail	Jordan	1

